# Extremely high expression of serum alpha-fetoprotein level of gastric adenocarcinoma: a rare case with an unexpected well-prognosis

**DOI:** 10.1186/s40064-016-3719-7

**Published:** 2016-12-01

**Authors:** Weihua Gong, Dawei Shou, Ping Gong

**Affiliations:** 1 Department of Surgery and Medicine, Second Affiliated Hospital of School of Medicine, Zhejiang University, Jiefang Road #88, Hangzhou City, 310009 Zhejiang Province People’s Republic of China; 2 Department of Oncology, First Affiliated Hospital of Shihezi University School of Medicine, Shihezi City, People’s Republic of China

**Keywords:** AFP, Metastasis, Gastric cancer

## Abstract

**Introduction:**

Alpha-fetoprotein (AFP)-producing gastric cancer is a relatively rare form of stomach malignancy. Patients with higher serum AFP level (>300 ng/mL) will have a poorer prognosis.

**Case description:**

In this study, we present a case of 77-year-old woman with an extremely high expression of serum AFP level (>10,000 ng/mL) of gastric cancer. Histological examination revealed gastric adenocarcinoma (Bormann III gastric tumor) invading into the serosal layer and vessel. After receiving a successful total gastrectomy with D2 dissection, this patient underwent chemotherapy with SOX [oxaliplatin (130 mg/m^2^/day) iv. at day 1 + S-1 (80 mg/m^2^/day) from day 1–day 14]. The patient remains alive without disease for 29 months after surgery. Serum AFP level decreased to normal range.

**Discussion and Evaluation:**

We have evaluated the level of AFP, and discussed the reason for the good prognosis for this patient.

**Conclusions:**

The good prognosis may be related with her early stage (N0 and without liver metastasis) and radical surgery.

## Background

Alpha fetoprotein (AFP) is an oncofetal glycoprotein mainly produced in the liver, yolk sac and gastrointestinal tract during fetal period (Gitlin et al. 1972). Although serum AFP level is apparently low in adults, it may significantly increase in the pathological conditions including yolk sac tumors, hepatocellular carcinoma, and cirrhosis and hepatitis (Purves et al. 1970). However, some other diseases can also be related with elevated levels of AFP, gastric cancer included. Gastric cancer is the fourth common cancer and the second common cause of cancer death worldwide (Sun et al. 2015). AFP-producing gastric cancer(AFPGC) is a relatively rare aggressive malignancy and its incidence is around 3% among all gastric cancers, which is reported by Bourrille et al. (1970) for the first time (Kono et al. 2002). Indeed, most AFP-producing GC patients can not survive for more than 1 year (Inagawa et al. 2001). In this present paper, we describe a rare case of an old woman suffering from gastric cancer with an extremely high expression of serum AFP level (>10,000 ng/mL). After receiving a successful total gastrectomy with D2 dissection and chemotherapy, this patient remains alive for 29 months. Serum AFP level of the patient strikingly decreased to 0.7 ng/mL without a rebound. To the best of our knowledge, this is the first successful case with extremely high expression of pre-operative serum AFP level and an unexpected good prognosis.

## Case report

A 77-year-old woman presented to the surgery department with complaints of 1-month history of upper abdominal pain and dizziness. She denied fevers, change in the color of the urine or stool, nausea or vomiting. Physical exam revealed Anemic conjunctiva but no enlarged lymph nodes such as Virchow lymph nodes. In laboratory tests, occult blood test of stool showed positive. Hematological test revealed anemia, as evidenced by a low level of hemoglobin (Hb), 4.4 g/dL. The serum level of AFP was remarkably elevated (>10,000 ng/mL), whereas the level of carbohydrate antigen 19-9 (CA19-9) and carcinoembryonic antigen (CEA) were within normal range.

**Fig. 1:**
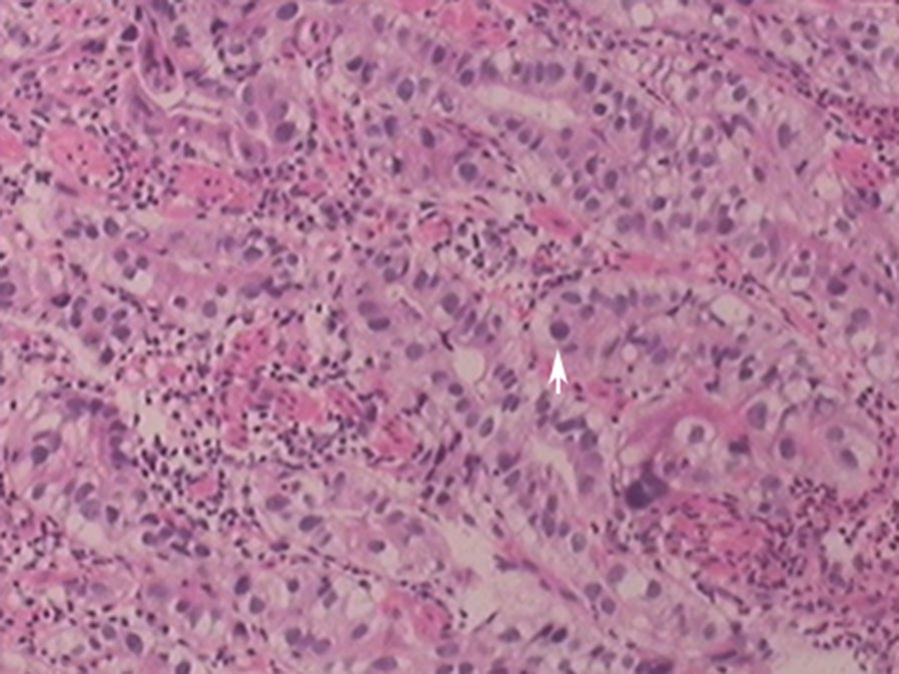
Haematoxylin and eosin staining for alpha-fetoprotein (AFP)-producing gastric adenocarcinoma. AFPGC cell (*white arrow*)

**Fig. 2:**
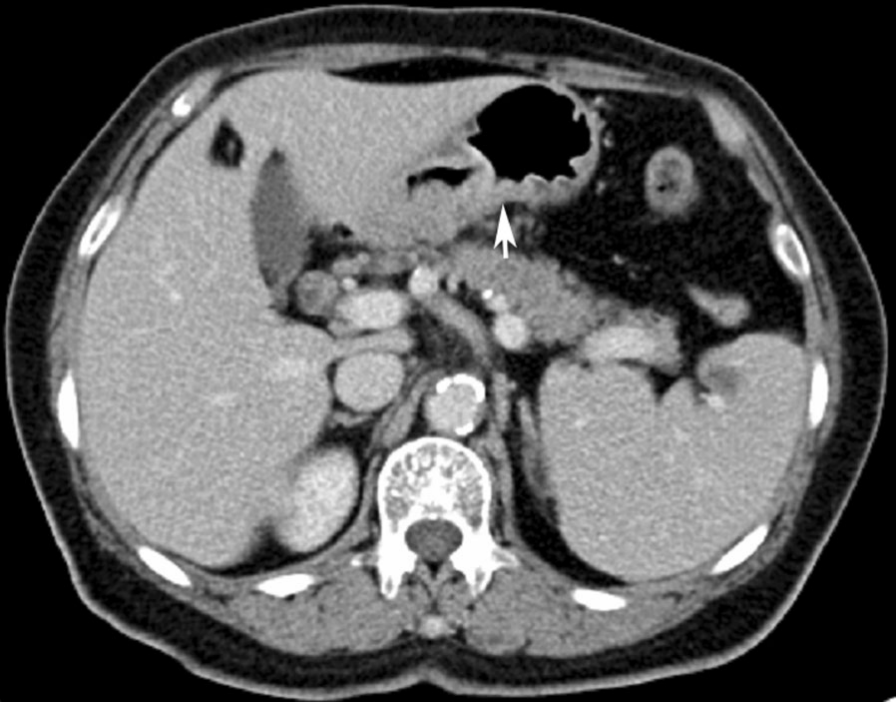
Computed tomography (CT) imaging of the abdomen before surgery showing a local thickened gastric wall in a 77-year-old woman. Thickening of the gastric antrum (*white arrow*)

Endoscopic examination revealed a protruding lesion (about 0.3 * 0.3 cm) in the inferior wall of corpus, patchy congestion of mucosa in the lesser curvature of corpus, and deep depression. It occupied the lumen about a quarter perimeter with infiltrated mucosa in the anterior wall of antrum. Gastric biopsy exhibited moderately differentiated adenocarcinoma (Fig. 1). To further evaluate the feature of the tumor and its surroundings, subsequent abdominal computed tomography (CT) scans were performed. The images manifested the thickened gastric antrum and the swelled lymph nodes surrounding the stomach (Fig. 2).

The patient was clinically diagnosed as AFP-producing gastric cancer. Afterwards, total gastrectomy with D2 lymph node dissection was performed. No liver or distant metastasis was observed. The resected specimen displayed that this tumor invaded into the serosal layer of gastric wall and vessel. Immunohistochemistry staining (IHC) showed AFP+++, hepatocyte−, CD34−, CD10−, CK8+++, CK18+++, CK19+++, CK20−, CK7−, P53 ++, and Ki67 50–60%. No metastasis of lymph nodes was found around the stomach.

**Fig. 3:**
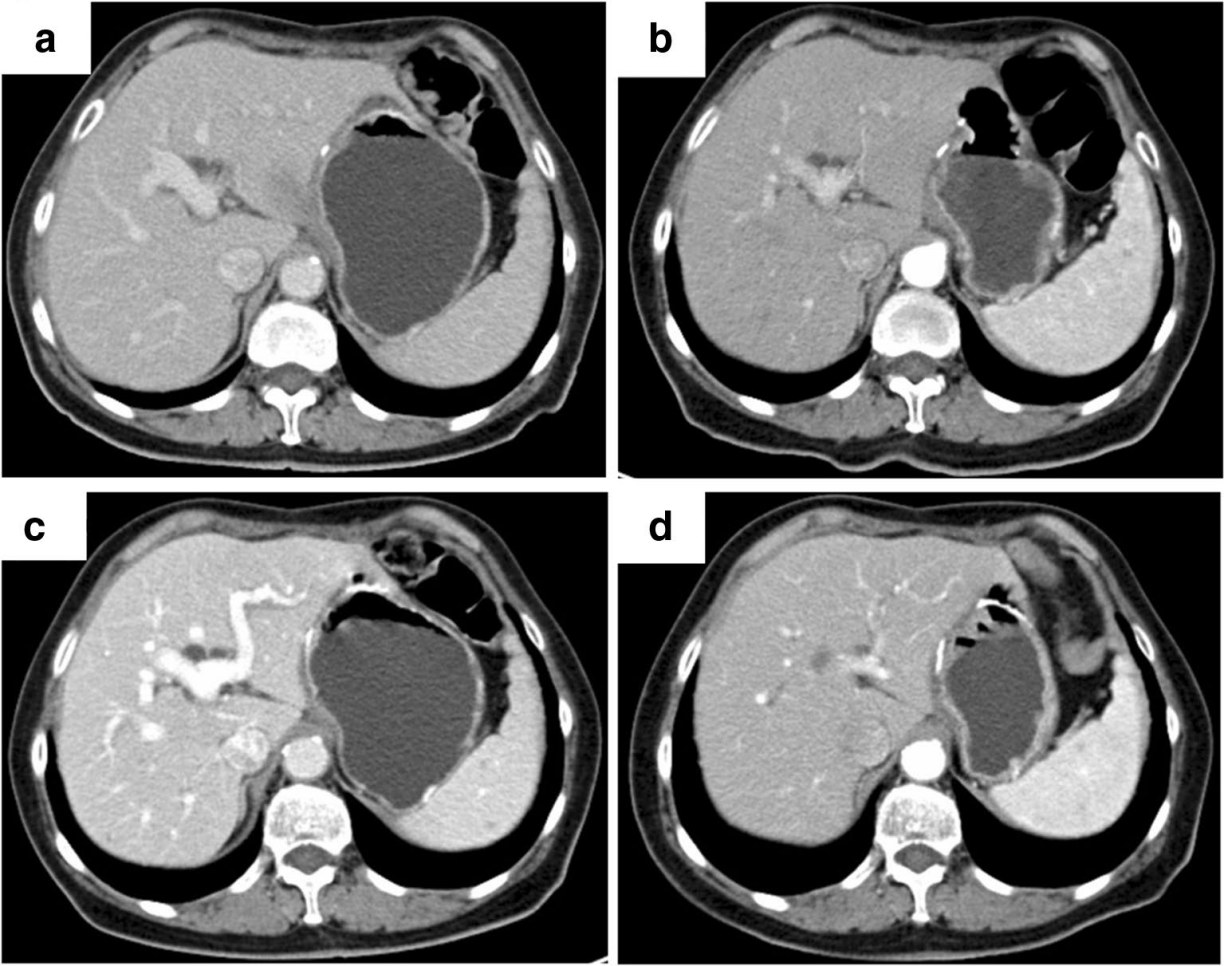
Abdominal computed tomography scans 6- (**a**), 12- (**b**), 18- (**c**), and 24- (**d**) months after surgical therapy. The scans do not exhibit any recurrence of the tumor or metastatic lymph nodes

**Fig. 4:**
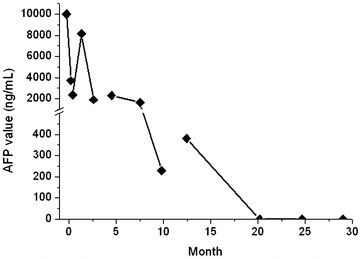
Serial alterations in serum alpha-fetoprotein (AFP) concentrations in a 77-year-old woman without recurrent AFP-producing gastric cancer during the entire 29-month post-operative course by far

Postoperatively, The patient successfully received six courses of SOX chemotherapy [oxaliplatin (130 mg/m^2^/day) iv. at day 1 + S-1 (80 mg/m^2^/day) from day day 1–day 14). Afterwards, a careful follow-up was made on the patient. Abdominal CT scan was made every 6 months (Fig. 3) and there is no evidence for recurrence or liver metastasis found. Test of tumor biomarkers was performed to monitor alteration of serum AFP level (Fig. 4). The patient has survived for 29 months and remains alive by far.

## Discussion

Since the first report of AFP-producing gastric cancer in 1970, AFP-producing gastric cancer has been classified into four histological subtypes: hepatoid, yolk sac tumor, enteroblastic and common adenocarcinoma type (Bourreille et al. 1970; Kinjo et al. 2012). Hepatoid adenocarcinoma of the stomach was reported by Ishikura et al. (1985) for the first time. The diagnosis of this type depends on morphology of hepatic differentiation, regardless of serum AFP levels (Nagai et al. 1993). Kinjo et al. (2012) suggest that AFPGC develops as common adenocarcinoma and enteroblastic type in the mucosa, and then differentiate into enteroblastic and hepatoid adenocarcinoma type. In this case, since no specific morphology was observed, we made the diagnosis of AFP-producing gastric cancer.

AFP-producing gastric cancer highly expresses VEGF-C, SALL4, and c-Met/HGF (Amemiya et al. 2000; Kamei et al. 2003; Ushiku et al. 2010). VEGF-C facilitates formation of rich neovascularization, and SALL4 maintains self-renewal and pluripotent properties of embryonic stem cells (Kamei et al. 2003; Ushiku et al. 2010). In the immunohistochemistry staining, Ki67 labeling index is significantly higher in the AFP-producing gastric cancers than in the AFP-negative gastric cancers. Ki67 is related with cell proliferation and tumor progression (Koide et al. 1999). Compared with AFP-negative gastric cancer, AFP-producing gastric cancer possesses higher proliferative activity, weaker apoptosis and richer neovascularization. In this case, Ki67 index is high, while the prognosis of the patient is well. IHC result is important but not the most significant factor determining the outcome of AFPGC.

The treatment of AFP-producing gastric cancer mainly includes radical surgery and chemotherapy. Radical surgery is of significance for AFPGC. Xie et al. (2015) have emphasized that R0 resection is the most important curative method. And there is a report that no significant difference of the prognosis exists between the AFP-positive group and the AFP-negative group after radical surgery (Nagai et al. 1993). The choice for chemotherapy is variable. Preoperative FLEP chemotherapy [a combination of chemotherapy with 5-fluorouracil (5-FU), leucovorin (LV), etoposide (VP-16) and Cis-diamminedichloroplatinum (CDDP)] was reported to improve the prognosis of AFP-producing GC owing to down-staging of cancer. A better response rate (70%) was observed among stage IV AFP-producing patients (Kochi et al. 2004). Combination of 5-FU and paclitaxel or combination of gemcitabine and cisplatin were also reported to show moderate efficacy (De Lange et al. 2004; Takeyama et al. 2007). Platinum is received by a large amount patients (Baek et al. 2011). In this case, R1 section and chemotherapy with SOX (oxaliplatin + S-1) was given.

The prognosis of AFP-producing gastric cancer is usually poor, which is due to advanced stage, liver metastasis (Kono et al. 2002; Shibata et al. 2007). The incidence of synchronous and metachronous liver metastasis is high in AFPGC patients (Hirajima et al. 2013; Inoue et al. 2010). Liver metastasis, can significantly affect the 5-year survival rate between patients with and without liver metastasis, which has been taken into account for the only independent prognostic factor (Hirajima et al. 2013). And if the patient is without lymph node and liver metastasis, they could always be cured (Xie et al. 2015). Apart from lymph node and liver metastasis, the level of serum AFP was reported to be associated with the poor prognosis despite no positive correlation. A significant poorer 1-, 3-, and 5-year survival was observed when serum AFP was >300 ng/mL (Lin et al. 2014). Despite the aggressive biological behavior of AFPGC, the proper treatment is a significant factor for the prognosis of AFPGC. Xie et al. (2015) proposed that R0 resection is of significance for the prognosis of AFPGC. In this case, the patient did not have lymph node and liver metastasis, though with high level of serum AFP, and R1 resection was performed followed by SOX chemotherapy. This may partly account for the good prognosis.

In addition, it is not well defined that alteration of pre-operative serum AFP level or post-operative serum AFP level can be utilized for predicting and monitoring gastric cancer recurrence (Inoue et al. 2010). Choi et al. (2006) reported that the serum AFP level at the time of initial diagnosis rather than at the time-point of tumor recurrence is strongly associated with subsequent recurrence event. In our present study, the initial level of serum AFP level was extremely high (>10,000 ng/mL) (Fig. 4). But this patient did not manifest tumor recurrence. Therefore, a large-scale research data is required for further investigation. Furthermore, owing to the limited amount of AFP-producing patients, it is difficult to sub-stratify them and make a comparison of prognosis among various pathological types. To date, no literature addressed this issue.

Taken together, AFP-producing gastric cancer is a rare type of gastric cancer, with an incidence of 3% among all gastric cancers. And AFP-producing gastric caner is not totally equal to hepatoid adenocarcinoma of the stomach. Though the prognosis of AFPGC is usually quite poor, it may be cured if the patient is in the early stage and R0 resection and chemotherapy are given. Early detection seems very important for those patients.

## Abbreviations

AFP: alpha-fetoprotein
AFPGC: AFP-producing gastric cancer
CA19-9: carbohydrate antigen 19-9
CEA: carcinoembryonic antigen
GC: gastric cancer
HGF: hepatocyte growth factor
IHC: immunohistochemistry staining
SALL4: Sall-like protein 4
VEGF-C: vascular endothelial growth factor-C
